# Evaluation of the Shape Memory Effect by Micro-Compression Testing of Single Crystalline Ti-27Nb Ni-Free Alloy

**DOI:** 10.3390/ma13010110

**Published:** 2019-12-25

**Authors:** Takashi Nagoshi, Takahisa Yasuda, Nao Otaki, Masaki Tahara, Hideki Hosoda, Masato Sone

**Affiliations:** 1 National Institute of Advanced Industrial Science and Technology, 1-2-1 Namiki, Tsukuba, Ibaraki 305-8564, Japan; 2 Institute of Innovative Research, Tokyo Institute of Technology, Yokohama 226-8503, Japan; yasuda.t.aee@gmail.com (T.Y.); otaki.8pn.nao@jp.nipponsteel.com (N.O.); tahara.m.aa@m.titech.ac.jp (M.T.); hosoda.h.aa@m.titech.ac.jp (H.H.); sone.m.aa@m.titech.ac.jp (M.S.); 3 CREST, Japan Science and Technology Agency, Yokohama 226-8503, Japan

**Keywords:** shape memory alloy, temperature variable micro-compression test, single crystal, biomedical alloy

## Abstract

In this work, micro-compression tests are performed at various temperatures with Ti-27Nb (at.%) single crystalline pillars to investigate anisotropic deformation behavior, including the shape memory effect. In non-tapered single-crystal pillars with loading directions parallel to [001], [011], and [111], transformation strain and stress show orientation dependence. [001]-oriented micropillars with aspect ratios of 2 and 1.5 demonstrate temperature-dependent transformation stress during micro-compression at various temperatures. Although more stress is required to induce martensite transformation in the pillar with the lower aspect ratio, the temperature dependence of ~1.8 MPa/K observed in both pillars is in good agreement with that of bulk Ti-27Nb.

## 1. Introduction

Shape memory is a property of materials in which materials can recover their shape even after plastic deformation. The unique properties of shape memory alloys originate from martensitic transformation. Shape recovery in these alloys occurs with unloading or heating [1]. The shape memory effect has been widely utilized in sensing, actuation, energy conversion, and smart devices [2,3]. Shape memory alloys are used in bone plates, coils, clips, and blood vessel stents owing to their superior shape memory properties, which include super-elasticity and corrosion resistance. Ti–Ni shape memory alloys are widely employed as biomedical materials. On the other hand, development of Ni-free biomedical shape memory alloys is important for biomedical applications due to concerns about Ni hypersensitivity [4,5].

Recent research has shown that β-type Ti–Nb binary alloys have attractive properties for a wide range of biomedical applications [6,7]. Ti–Nb alloys retain the β phase at room temperature when they contain sufficient amounts of the β-stabilizing element Nb. The shape memory effect in these materials originates in martensitic transformation from the body-centered cubic β phase to C-centered orthorhombic α” martensite, which can be transformed back to the β phase by unloading or heating. The shape memory properties of Ti–Nb-based alloys have been investigated in various studies, and the effects of alloying [8,9], thermomechanical treatment [10,11], and interacting deformations [12,13] on shape memory have been evaluated. However, the anisotropic features of their mechanical properties have not been well documented due to the difficulty of single crystal fabrication. For a better understanding of the nature of Ti–Nb shape memory alloys, it is very important to investigate their mechanical properties using single crystalline pillars. In addition, the mechanical behavior of a sample at the microscale is different from that of the bulk material because the strength of a metal increases as sample size decreases [14,15]. The effect of sample size on the shape memory of alloys has also been a topic of interest for applications in microelectromechanical systems (MEMS). Recent experimental studies have revealed a wide range of influences that sample size has on the shape memory effect [16,17,18]. In the present work, the shape memory effect in Ti–27Nb single crystals at scales relevant for MEMS applications was investigated using micro-compression testing at various temperatures.

## 2. Materials and Methods

Ti–27Nb (at.%) alloy ingots were prepared by arc melting with a copper crucible and tungsten electrode in an argon atmosphere. Ingots were solution treated at 1173 K for 1 h in an argon atmosphere followed by water quenching, and the oxidized surface layer that formed by water quenching was removed by chemical etching at 333 K with a solution of HF:HNO_3_:H_2_O = 7:8:10 in volume. Then, the ingots were mechanically thinned to a thickness of 0.1 mm. Compression micropillars were fabricated at the edges of thin plates secured in a custom-built aluminum holder which could be easily transferred for SEM, focused ion beam (FIB) modification, and experiments with a mechanical testing machine. Non-tapered pillars were formed by ion irradiation vertical to the pillar axis using a FB2001 FIB (Hitachi, Tokyo, Japan). Details of the fabrication method can be found elsewhere [19,20]. Two square sections were milled from each pillar by ion irradiation normal to the plate, leaving a rectangle pillar on the plate edge. The corners of the pillar were then removed by irradiation at 45° angles from the plate to form a smaller square pillar. To obtain a smooth surface, damaged areas on each face of the pillar were removed by irradiation with a low-current beam. The final dimensions of the pillars fabricated for micro-compression tests were 10 × 10 × 20 µm^3^, and additional pillars 9 × 9 × 13.5 µm^3^ in size were fabricated for the temperature-variable micro-compression tests. Measurements of the crystal orientations of the pillars were performed before and after fabrication with a S-4300SE SEM (Hitachi, Tokyo, Japan) equipped with an e-Flash electron back-scatter diffraction (EBSD) detector (Bruker AXS GmbH, Karlsruhe, Germany) to obtain the desired orientations of [111], [011], and [001].

**Figure 1:**
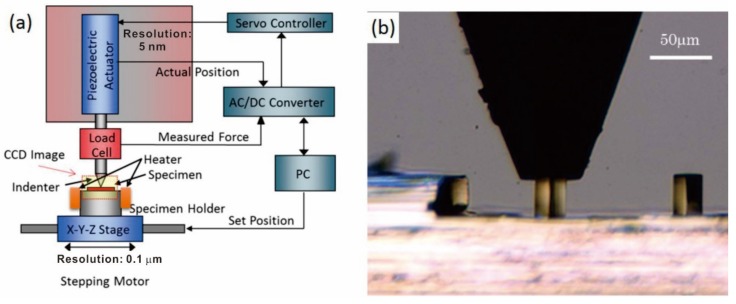
(**a**) Schematic illustration of the temperature-variable micro-compression setup and (**b**) image captured with a charge-coupled device (CCD) camera during in situ compression.

Micro-compression tests were conducted with a test machine developed by our group [19,20]. A schematic diagram of the machine is shown in Figure 1a. The specimen holder attached to the X-Y-Z stage was carefully manipulated to align each pillar with a flat-ended indenter, which was viewed with a charge-coupled device (CCD) camera (WRAYMER, Osaka, Japan). A piezoelectric actuator equipped with an indenter and a load cell was used to apply displacement force at a rate of 0.05 µm/s. Applied displacement and load were recorded with an AC/DC converter every 33 ms. In situ optical images were obtained with the CCD camera, as shown in Figure 1b. For temperature-variable micro-compression, the sample holder equipped with a thermocouple was sandwiched between two aluminum nitride heaters. Repeated loading and unloading cycles were performed and the temperature was increased by 10 K for each cycle. A sufficient waiting period during each cycle allowed the temperature to stabilize until the highest testing temperature of 393 K was reached.

## 3. Results and Discussion

The compositions of the solution-treated samples were determined with inert gas fusion and inductively coupled plasma analysis with a measurement error of less than 0.3% for each analysis. The material compositions are shown in Table 1. The Nb concentration was very close to the target amount and only low concentrations of other elements were observed. The martensitic transformation start temperature, *M_s_*, at this Nb concentration was estimated to be ~230 K in a previous study [21]. Thus, martensitic transformation and recovery were expected at all temperatures used for the compression tests, including room temperature.

**Figure 2:**
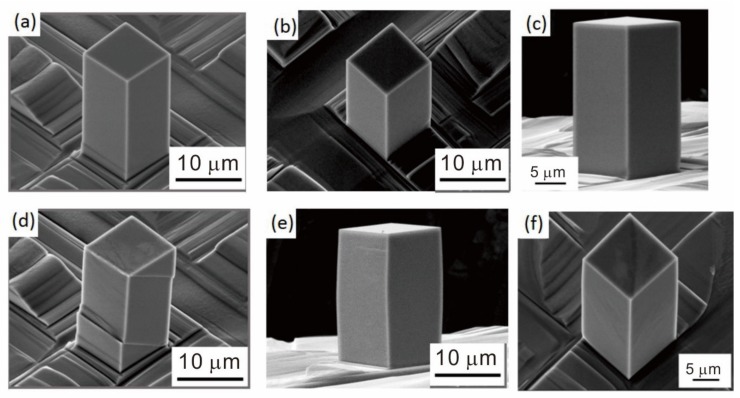
SEM images of the pillars before (**a**–**c**) and after (**d**–**f**) the micro-compression test. Orientations of the specimens: (**a**,**d**) [111]; (**b**,**e**) [011]; and (**c**,**f**) [001].

**Figure 3:**
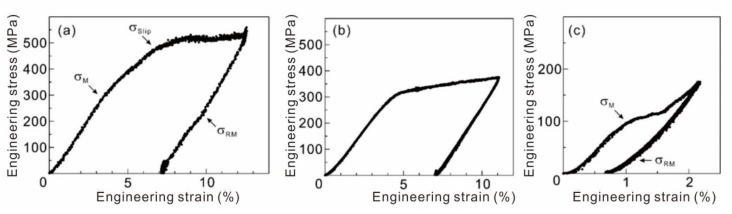
Engineering stress–strain curves of the single crystalline pillars with orientations (**a**) [111], (**b**) [011], and (**c**) [001].

SEM images of the micro-compression samples with different orientations collected before and after compressive deformation are shown in Figure 2. The pillars had smooth surfaces and were not tapered due to the fabrication method with FIB milling from vertical to pillar axis. The engineering stress–strain curves at orientations of [111], [011], and [001] parallel to the loading axis are shown in Figure 3. The deformation behavior of the pillars differed significantly depending on their crystal orientation. Transformation strain during martensitic transformation from the β to the α” phase was calculated from changes in the lattice constants. Based on a previous study by Kim et al. [21], the lattice constants of the β phase (a_0_) and the orthorhombic α” phase (*a*’, *b*’, *c*’) were calculated from Nb concentration of samples and were found to be a_0_ = 0.3290 nm, *a*’ = 0.3225 nm, *b*’ = 0.4770 nm, and *c*’ = 0.4615 nm. Considering the correspondence between the β and α” phase lattices, the maximum possible transformation strains during compression at orientations of [111], [011], and [001] were calculated to be 1.2%, 0.8%, and 2.0%, respectively. Plastic strain exceeded the maximum possible transformation strain during deformation of the [011]-oriented pillar, which implied that martensitic transformation did not occur or was mixed with deformation by slip activation. SEM observation following deformation of the [011]-oriented pillar did not reveal any trace of martensitic transformation. The [111]-oriented pillar yielded with martensitic transformation at transformation start stress, σM and deformed at stress for activation of dislocation slip (σSlip). Martensite recovered at transformation recover stress (σRM) with unloading. Less than 1% of the strain was generated from martensitic transformation and further recovered. Low σM was observed in the [001]-oriented pillar, which was unloaded before slip deformation could occur. The transformation strain was about 1%, and partial recovery was observed. The transformation strain should be smaller than the maximum value during pillar compression because transformation is limited to the body of the pillar. This is illustrated clearly in Figure 2d. The middle of the pillar was transformed and traces of the post-transformation planes are visible at each end. Taking the non-transformed areas at the top and bottom of the pillar into consideration, the calculated transformation strain and the observed strain during martensitic transformation are in good agreement.

**Figure 4:**
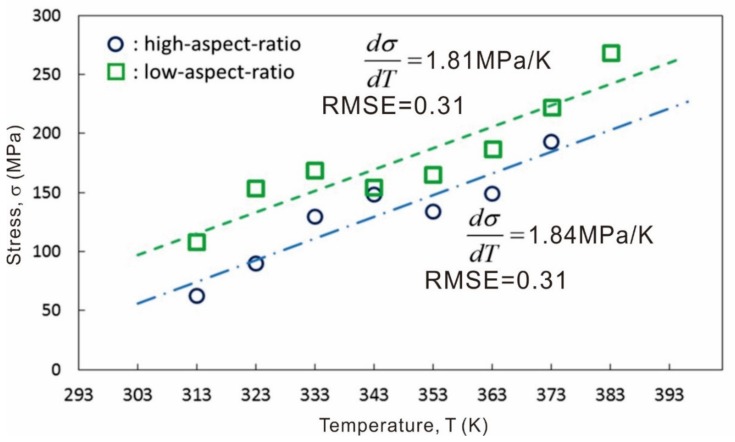
Dependence of transformation stress on temperature during micro-compression. Legend: RMSE, root mean square error.

Temperature-variable micro-compression tests were performed with the [001]-oriented sample, which clearly yielded to transformation at a low stress of ~100 MPa. Transformation stress, *σ* (MPa), was recorded as the stress at initial yielding in each loading and unloading cycle. Transformation stress has been plotted according to testing temperature in Figure 4. Transformation stress was found to be higher in pillars with an aspect ratio of 1.5, which may have been due to the larger impact of lateral stress at the tops of the pillars from the contacting indenter. However, the relationship between transformation stress and testing temperature at both aspect ratios was almost identical. The stress required for martensitic transformation reflected the thermodynamic stability of the β phase, which is undisputedly thought to obey the well-known Clausius-Clapeyron relationship, i.e.:

(1) $$\frac{\Delta \sigma }{\Delta T}=-\frac{\rho \Delta S}{\varepsilon_{M}}=-\frac{\rho \Delta H}{T\times \varepsilon_{M}}$$

Here, *ρ* is the density of the transformation phase, Δ*S* and Δ*H* are the changes in entropy and enthalpy during transformation, respectively, and *ε_M_* is the maximum transformation strain. These parameters are constant in a given alloy system with the exception of transformation strain, which depends on the direction of stress relative to the crystal orientation. Al-Zain et al. [22] have reported the dependence of transformation stress on temperature in the Ti–27Nb alloy as 2 MPa/K. This value is slightly larger than the value of 1.8 MPa/K with a root mean square error (RMSE) of 0.3 obtained in this work for compression of the [001]-oriented single crystal. However, considering the difference in transformation strain, the dependence of transformation strain on temperature observed in the present study is in good agreement with the bulk value. The difference in transformation strain would also be responsible for the orientation-dependent transformation stress observed during single-crystal pillar compression. Higher transformation stress in the [111] pillar was due to the lower transformation strain in the [111] orientation.

## 4. Conclusions

In this study, we investigated the shape memory effect of a Ti–27Nb biomedical alloy. Non-tapered single-crystal micropillars were fabricated by FIB and underwent compression testing with a custom-designed, variable temperature micro-compression system. Anisotropic deformation behavior was observed during compression at room temperature in pillars with orientations of [001], [011], and [111]. Changes in strain and stress with martensitic transformation were explained well by the calculated transformation strains for the given loading directions. Taking into account the specimen orientation, the dependence of transformation strain on temperature was in good agreement with the bulk value. The shape memory effect in Ti–27Nb at scales relevant for MEMS applications was consistent with its bulk properties.

## Figures and Tables

**Table 1:** Compositions of the Ti–27Nb samples.

Elements	Ti	Nb	Fe	Ni	Cu	Mo	W	Au	O	H	N
Measured composition (at.%)	Bal.	26.9	0.0519	0.0988	0.141	0.0302	0.0221	0.0118	0.119	0.36	0.0936
